# Supplementary material for: Specific Effects of Trabectedin and Lurbinectedin on Human Macrophage Function and Fate—Novel Insights
Source: Cancers (Basel). 2020 Oct 20;12(10):3060. doi: 10.3390/cancers12103060 (PMC7590144; doi:10.3390/cancers12103060)
Supplement: Supplementary file 1 [file cancers-12-03060-s001.pdf]

Supplementary Materials: Specific Effects of Trabectedin and Lurbinectedin on Human Macrophage Function and Fate—Novel Insights

Adrián Povo-Retana, Marina Mojena, Adrian B. Stremtan, Victoria B. Fernández-García, Ana Gómez-Sáez, Cristina Nuevo-Tapioles, José M. Molina-Guijarro, José Avendaño-Ortiz, José M. Cuezva, Eduardo López-Collazo, Juan F. Martínez-Leal and Lisardo Boscá

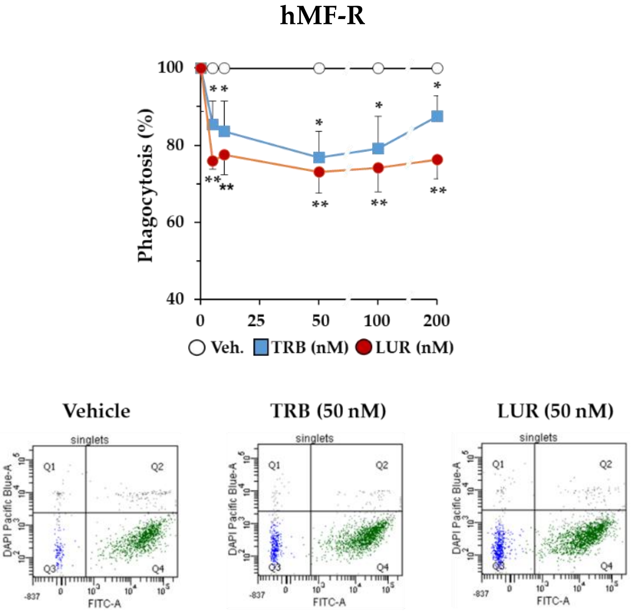

Figure S1. Treatment of hMF with TRB and LUR attenuates zymosan-dependent phagocytosis. hMF-R cells were treated for 24h with the indicated concentration of TRB and LUR and the incorporation of FITC-zymosan was determined by flow cytometry. Results show the means ± S.E.M. of 6 independent donors and representative flow cytometry plots. * $p < 0.05$; ** $p < 0.01$ *vs*. the vehicle (Veh.) condition.

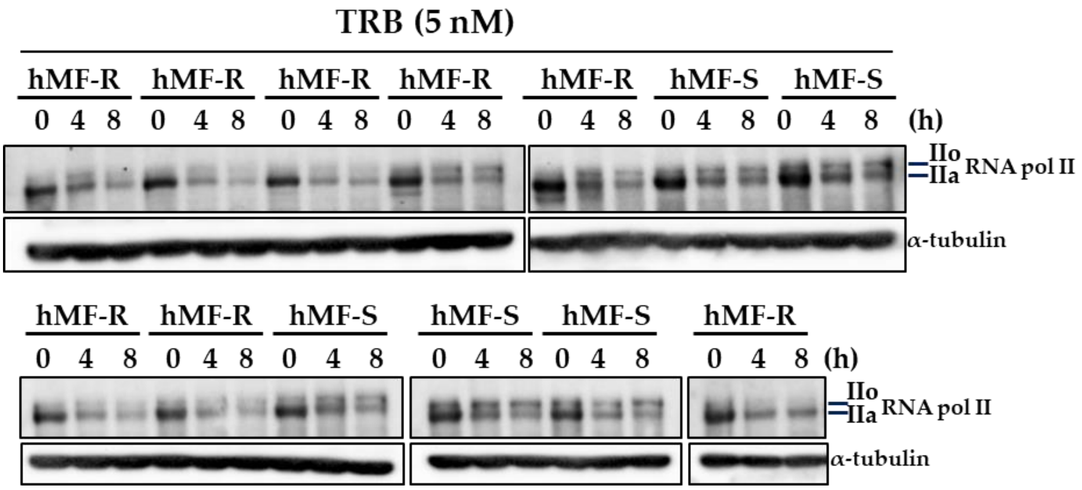

Figure S2. Treatment of hMF with TRB promotes a time-dependent dephosphorylation of RNA pol II, irrespective of the effects of the drug on cell viability.hMF-S and hMF-R cells were treated with 5 nM TRB and, at the indicated times, samples were processed and the levels of RNA pol II were determined by immunoblot. IIo, hyperphosphorylated RNA pol II; IIa, dephosphorylated RNA pol II.

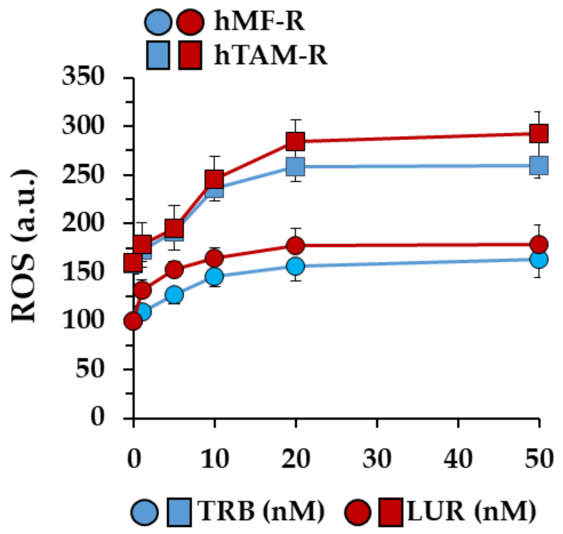

Figure S3. Dose-dependent effect of ROS production after the treatment of hMF-R and hTAM-R with TRB and LUR attenuates. Cells were treated for 24 h with the indicated concentrations of the drugs and the ROS production was determined by flow cytometry, following the oxidation of DCFH. Results show the means + S.E.M. of 6 independent donors. a.u.; arbitrary units.

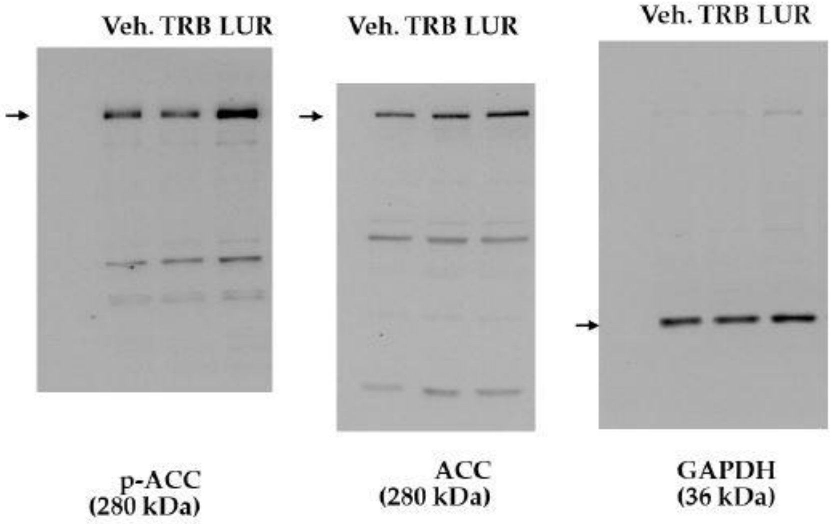

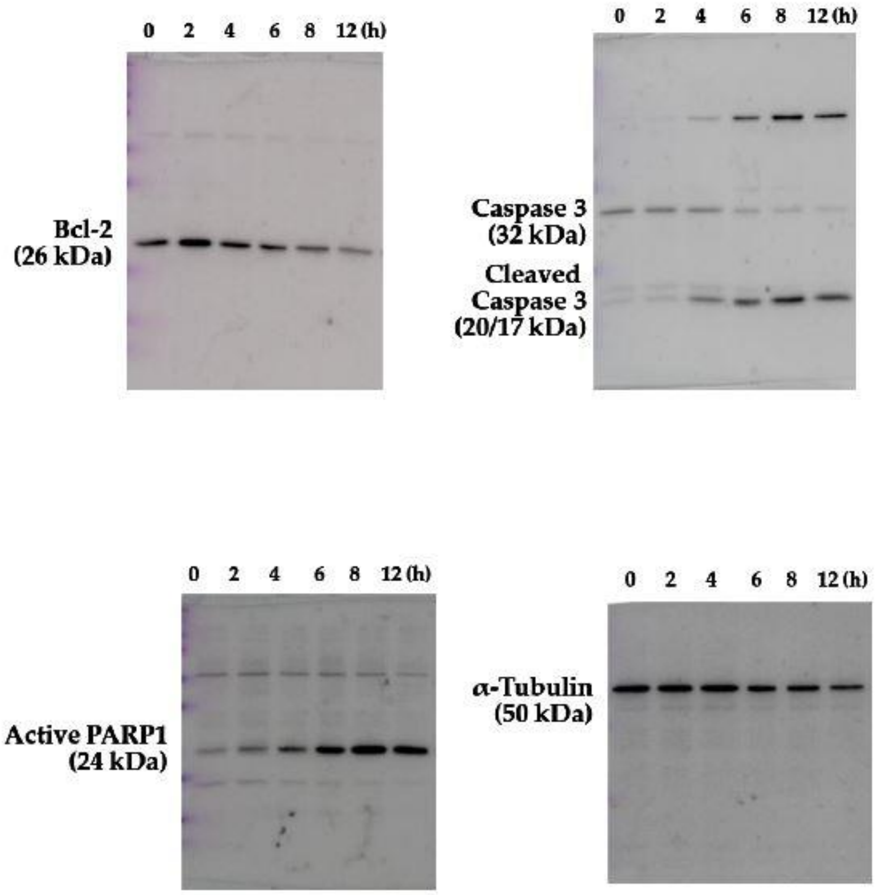

Figure S4. Uncropped Western Blot Images.

Publisher's Note: MDPI stays neutral with regard to jurisdictional claims in published maps and institutional affiliations.

© 2020 by the authors. Licensee MDPI, Basel, Switzerland. This article is an open access article distributed under the terms and conditions of the Creative Commons Attribution (CC BY) license (http://creativecommons.org/licenses/by/4.0/).
